# Topographic Organization of Glutamatergic and GABAergic Parvalbumin-Positive Neurons in the Lateral Habenula

**DOI:** 10.1523/ENEURO.0069-24.2024

**Published:** 2024-07-12

**Authors:** Thi Van Trang Nguyen, Tomoya Nakamura, Hiroyuki Ichijo

**Affiliations:** Department of Anatomy, Faculty of Medicine, University of Toyama, Sugitani 2630, Toyama 930-0194, Japan

**Keywords:** GABAergic, glutamatergic, lateral habenula, parvalbumin, topography

## Abstract

Parvalbumin-expressing (PV) neurons, classified by their expression of the calcium-binding protein parvalbumin, play crucial roles in the function and plasticity of the lateral habenular nucleus (LHb). This study aimed to deepen our understanding of the LHb by collecting information about the heterogeneity of LHb PV neurons in mice. To achieve this, we investigated the proportions of the transmitter machinery in LHb PV neurons, including GABAergic, glutamatergic, serotonergic, cholinergic, and dopaminergic neurotransmitter markers, using transcriptome analysis, mRNA in situ hybridization chain reaction, and immunohistochemistry. LHb PV neurons comprise three subsets: glutamatergic, GABAergic, and double-positive for glutamatergic and GABAergic machinery. By comparing the percentages of the subsets, we found that the LHb was topographically organized anteroposteriorly; the GABAergic and glutamatergic PV neurons were preferentially distributed in the anterior and posterior LHb, respectively, uncovering the anteroposterior topography of the LHb. In addition, we confirmed the mediolateral topography of lateral GABAergic PV neurons. These findings suggest that PV neurons play distinct roles in different parts of the LHb along the anteroposterior and mediolateral axes, facilitating the topographic function of the LHb. It would be interesting to determine whether their topography is differentially involved in various cognitive and motivational processes associated with the LHb, particularly the involvement of posterior glutamatergic PV neurons.

## Significance Statement

The parvalbumin-expressing (PV) neurons are key in the understanding of the neural circuits in the lateral habenula (LHb). There are three subsets of LHb PV neurons in mice: glutamatergic, GABAergic, and double-positive for both glutamatergic and GABAergic machinery, with the majority being glutamatergic. Herein, we demonstrate that these subsets of the LHb PV neurons were topographically organized anteroposteriorly, suggesting topographic function in the LHb.

## Introduction

The habenula is situated in the dorsal diencephalon, which comprises the dorsal–medial end of the thalamus in mammals, and consists of the medial and lateral nuclei (Herkenham and Nauta, 1977, 1979; Hikosaka, 2010; Aizawa et al., 2012). The lateral habenular nucleus (LHb) receives various afferents from the limbic forebrain, basal ganglia, and medial prefrontal cortex (Herkenham and Nauta, 1977, 1979; Kim and Lee, 2012; Yang et al., 2018). Efferents from the LHb project to the midbrain monoaminergic centers, including the dopaminergic nuclei (ventral tegmental area and substantia nigra pars compacta; Herkenham and Nauta, 1979; Christoph et al., 1986; Ji and Shepard, 2007; Lecourtier and Kelly, 2007; Jhou et al., 2009), serotonergic nuclei (dorsal and median raphe nuclei; Herkenham and Nauta, 1977; Wang and Aghajanian, 1977), and GABAergic nucleus (rostromedial tegmental nucleus; Jhou et al., 2009; Kaufling et al., 2009). The LHb plays an important role in various cognitive and motivational processes through its action as a hub between emotions and behavior. Furthermore, it encodes unpleasant rewards and is associated with psychiatric diseases, such as depressive disorders (Matsumoto and Hikosaka, 2007, 2009; Nuno-Perez et al., 2018; Yang et al., 2018). In terms of the number of parvalbumin-expressing (PV) neurons (Webster et al., 2020), elaboration of perineuronal nets (Bernard and Prochiantz, 2016; Bradshaw et al., 2018; Nakamura et al., 2021), and neuronal activity under stress (Tchenio et al., 2017; Yang et al., 2018), the mouse LHb matures through four stages: postnatal days (P) P1–9, P10–20, ∼P35, and afterward. The second stage of maturation, from P10 to P20, is crucial because early life stress in this stage specifically results in late effects in adulthood: fewer PV neurons in the LHb and anxiety- and depression-like behaviors (Nakamura et al., 2021), showing that the plasticity of LHb maturation depends on experiences. This suggests that PV neurons are involved in LHb maturation.

PV neurons play key roles in regulating the plasticity of neuronal circuits (Bernard and Prochiantz, 2016; Bradshaw et al., 2018). They are generally known as GABAergic fast-spiking neurons in the cortex (Celio, 1986; del Rio et al., 1994; Tamamaki et al., 2003; Markram et al., 2004; Rudy et al., 2011) and control plasticity in the visual cortex (Sugiyama et al., 2008, 2009; Chittajallu and Isaac, 2010; Kuhlman et al., 2013; Takesian and Hensch, 2013). Given this role, it is important to determine whether the PV neurons are inhibitory interneurons in the mouse LHb. In contrast, glutamatergic PV neurons have been reported in the hypothalamus (Faget et al., 2018; Kisner et al., 2018; Roccaro-Waldmeyer et al., 2018; Tooley et al., 2018; Siemian et al., 2020; Laing et al., 2022) and many other brain regions (Xu et al., 2022). Webster et al. (2020) showed that *pv* mRNA-positive neurons express *vgat* and *vglut2* mRNA. Another previous study also showed that ∼60% of LHb PV neurons exhibit GABA immunoreactivity (Nakamura et al., 2021). These studies have indicated that LHb PV neurons are heterogeneous in their neurotransmitter machinery and are distributed differently in the medial and lateral LHb. However, their details have not been thoroughly elucidated.

To clarify the characteristics of PV neurons in the mouse LHb, in the present study, we comprehensively investigated LHb PV neurons in terms of the proportion of the transmitter machinery: GABAergic, glutamatergic, serotonergic, cholinergic, and dopaminergic neurotransmitter markers using transcriptome analysis, mRNA in situ hybridization chain reaction (HCR; Choi et al., 2018), and immunohistochemistry (IHC). By elucidating the expression of the transmitter machinery in the PV neurons and quantitatively comparing their composition, we examined the overall topographic organization of the PV neurons in the LHb. Through this analysis, we demonstrated that the LHb is topographically organized anteriorly and mediolaterally.

## Materials and Methods

### Animals

All experimental procedures conducted on animals in this study complied with the ARRIVE guidelines. This study was performed in strict accordance with the Guidelines for the Care and Use of Laboratory Animals approved by the University of Toyama and US National Institutes of Health Guide for the Care and Use of Laboratory Animals. The study design was approved by the Ethics Committee for Animal Experiments at the University of Toyama (license numbers: A2019MED-34 and A2022MED-2). Wild-type male and female C57BL/6J mice were purchased from Japan SLC. All mice were housed in a temperature-controlled room (22–25°C) under a 12/12 h light/dark cycle (lights were turned on at 05:00 and off at 17:00). Food (CE-2; CLEA Japan) and water were provided *ad libitum*. Experiments were performed on mice at age 60–70 d. As separate analyses of males and females yielded similar results, male and female data were pooled and analyzed together. For all analyses, data from five mice (*N *= 5) were used, except for the colocalization analysis of *vglut2* and *gad2* in the LHb (*N *= 4) and *vglut1* and *gad1* in the cingulate cortex (*N *= 3).

### Analysis of single-cell RNA sequencing data of *pv* neurons in the LHb

The single-cell RNA sequencing data used in this study have been published previously (Hashikawa et al., 2020) and are accessible from the NCBI for Biotechnology Information Gene Expression Omnibus (accession number: GSE137478). Using Seurat V5.03 (Hao et al., 2024), we followed commands (https://github.com/stuberlab/Hashikawa-Hashikawa-2020/tree/master) and cluster classification methods such as those used by Hashikawa et al., employing canonical markers for neuronal cells (Macosko et al., 2015; Wu et al., 2017; Saunders et al., 2018; Zeisel et al., 2018) such as Stmn2 and Thy1, and markers for LHb neurons, such as Pcdh10, Htr2c, and Gabra1, to cluster the LHb neurons (Hsu et al., 2014; Mohan et al., 2016; Hashikawa et al., 2020). As we used a newer version of Seurat compared with that used by Hashikawa et al. (2020), the LHb clusters in our data were further subdivided. We used Seurat's FeatureScatter to plot the log-transformed and normalized gene expression levels of each cell in a scatter plot and examined the expression of glutamatergic markers (*vglut1*, *vglut2*, and *vglut3*) and GABAergic markers (*gad1*, *gad2*, *vgat*, and *gat*) in *pv* neurons. Data were scaled and centered; thus, genes with expression levels below the mean were assigned negative values.

### Preparation of brain sections

Mice were deeply anesthetized through intraperitoneal injection of a combination of 0.3 mg/kg medetomidine (Nippon Zenyaku Kogyo), 4.0 mg/kg midazolam (Astellas Pharma), and 5.0 mg/kg butorphanol (Meiji Seika Pharma). Subsequently, mice were transcardially perfused with phosphate-buffered saline (PBS) followed by 4% paraformaldehyde (Nacalai Tesque) in PBS. Mouse brains were removed and postfixed overnight in the same fixative at 4°C. Brains were coronally sectioned into 40 µm slices using a vibratome (Leica VT1000S; Leica Microsystems Nussloch). These sections were further fixed in 4% paraformaldehyde in PBS for 2 h at 4°C. Subsequently, they were rinsed in PBS three times for 10 min at room temperature and stored in 70% ethanol at 4°C until staining. Approximately 30 sections were serially obtained along the length from the anterior (at −0.95 mm from the bregma) to the posterior (at −2.15 mm from the bregma) region, including the LHb, basolateral amygdala, CA3 region of the hippocampus, and the cingulate cortex (Franklin and Paxinos, 2007). Serial sections from the anterior to posterior were assigned to alternative five groups (the alternative sections), and the sections were spaced 160 µm apart.

### HCR mRNA fluorescence in situ hybridization

HCR mRNA fluorescence in situ hybridization (HCR mRNA-FISH) was performed according to the protocol described by Choi et al. (2018). DNA probe sets, DNA HCR amplifiers, hybridization buffer, wash buffer, and amplification buffer were purchased from Molecular Instruments. All probes used in this study are shown in Table 1. Detection was performed under RNase-free conditions. Brain sections were incubated in 8% sodium dodecyl sulfate (Fujifilm Wako Pure Chemical) in PBS for 2 h at room temperature, after which they were rinsed three times for 1 h with 2× sodium–saline citrate (SSC) buffer stock solution (Nacalai Tesque) at room temperature and subsequently incubated in the probe hybridization buffer (Molecular Instruments) for 5 min at 37°C. After removing the probe hybridization buffer, the hybridization buffer with 4 nmol/l probe mixture (probe solution; Molecular Instruments) was added, and the sections were incubated overnight (16–20 h) at 37°C.

**Table 1.** Details of probes for in situ HCR

Numbers	Probes	Target gene	HCR amplifier	Fluorophore
1	*vglut1*	Slc17a7	B1	Alexa 594
			B4	Alexa 488
2	*vglut2*	Slc17a6	B1	Alexa 594
			B4	Alexa 488
3	*vglut2(set1)*	Slc17a6_set1	B1	Alexa 594
4	*vglut2(set2)*	Slc17a6_set2	B4	Alexa 488
5	*vglut3*	Slc17a8	B4	Alexa 488
6	*gad1*	Gad1	B1	Alexa 594
7	*gad2*	Gad2	B4	Alexa 488
8	*gad2(set1)*	Gad2_set1	B1	Alexa 594
9	*gad2(set2)*	Gad2_set2	B4	Alexa 488
10	*vgat*	Slc32a1	B1	Alexa 594
11	*parvalbumin*	Pvalb	B4	Alexa 488
12	*serotonin transporter*	Slc6a4	B1	Alexa 594
13	*gat*	Slc6a1	B1	Alexa 594
14	*enhanced green fluorescent protein*	EGFP	B1	Alexa 594
15	*choline acetyltransferase*	ChAT	B4	Alexa 488
16	*tyrosine hydroxylase*	Th	B4	Alexa 488

The DNA probe set size, HCR amplifier, fluorophore, and for each target are shown.

RNase-free conditions were not maintained from the amplification process. Probes were removed by washing brain sections four times for 15 min each with the HCR probe wash buffer (Molecular Instruments) at 37°C. The sections were then rinsed two times for 5 min with 5× SSC containing 0.1% polyoxyethylene 20 sorbitan monooleate (Tween 20, Fujifilm Wako Pure Chemical; 5× SSCT) at room temperature. Sections were incubated in an amplification buffer (Molecular Instruments) for 5 min at room temperature. Before using the hairpins (Molecular Instruments), samples were heated to 95°C for 90 s, cooled on ice, and placed in the dark at room temperature for 30 min (snap-cooled hairpins). Sections were incubated in an amplification buffer containing 60 nmol/l snap-cooled hairpins for 24 h in the dark at room temperature. The sections were washed with 5× SSCT in the dark at room temperature for 5 min, twice for 30 min, and twice for 5 min.

The following DNA probe sets were used: *gad1* (Gad1), *gad2* (Gad2), *vgat* (Slc32a1), *gat* (Slc6a1), *vglut1* (Slc17a7), *vglut2* (Slc17a6), *vglut3* (Slc17a8), *parvalbumin* (Pvalb), *serotonin transporter* (Slc6a4), *choline acetyltransferase* (ChAT), and *tyrosine hydroxylase* (Th; Molecular Instruments).

As positive controls for HCR mRNA-FISH, we separated the probe sets of *vglut2* and *gad2* into two sets, each with a different HCR amplifier and fluorophore. The DNA probe sets used included *vglut2* (Slc17a6_set1), *vglut2* (Slc17a6_set2), *gad2* (Gad2_set1), and *gad2* (Gad2_set2; Molecular Instruments), and identical signals were obtained. As a negative control, experiments were performed with an *enhanced green fluorescent protein* (EGFP; Molecular Instruments), and no signals were obtained. These results confirm the specificity of HCR.

### IHC

IHC was performed following HCR mRNA FISH. The sections were rinsed in PBS containing 0.5% Triton X-100 (Fujifilm Wako Pure Chemical; PBT), blocked with 3% bovine serum albumin (#9048-46-8; Fujifilm Wako Pure Chemical) in PBT, after which they were incubated for 3 d with the primary antibody in 3% bovine serum albumin in PBT. The following primary antibodies were used: anti-parvalbumin mouse antibody (#P3088, 1:10,000 dilution; Sigma-Aldrich), anti-parvalbumin goat antibody (#AB_2571614, 1:500 dilution for double staining with tryptophan hydroxylase, Frontier Institute), anti-tryptophan hydroxylase mouse antibody (#T-0678, 1:500 dilution; Sigma-Aldrich), anti-choline acetyltransferase goat antibody (#AB144P, 1:1,000 dilution; Merck Millipore), and anti-tyrosine hydroxylase rabbit antibody (#OPA1-04050, 1:500 dilution; Thermo Fisher Scientific). The sections were then incubated with the appropriate secondary antibodies conjugated with Alexa 488, 594, or 647 for 2 h (1:200 dilution; Thermo Fisher Scientific), counterstained with 4′,6′-diamidino-2-phenylindole dihydrochloride (1:10,000 dilution; D9542, Sigma-Aldrich), and mounted on glass slides with Mowiol 4-88 (Merck Millipore).

### Obtaining and analyzing images

Images were obtained using a confocal laser scanning microscope (LSM780 and LSM900; Zeiss). We obtained images of 708.49 × 708.49 µm with 1,024 × 1,024 pixels (LSM 780) and images of 820.63 × 820.63 µm with 1,024 × 1,024 pixels (LSM 900) using 20× objective lens. We obtained images of 1,416.99 × 1,416.99 µm with 1,024 × 1,024 pixels (LSM 780) and images of 1,277.8 × 1,277.8 µm with 1,024 × 1,024 pixels (LSM 900) with 10× objective lens. The LHb was found in six alternative sections, and cells positive for each cell type marker were counted from the anterior to the posterior. To analyze the anteroposterior differences in the LHb, the first and middle sections were omitted, and the remaining sections were divided into two anterior (−1.15 to −1.55 from the bregma) and two posterior sections (−1.75 to −2.15 from the bregma). The anterior part includes the anterior, central, marginal, parvocellular, and superior parts of the medial division of the lateral habenular complex and the magnocellular, marginal, oval, and parvocellular parts of the lateral division of the lateral habenular complex, as defined by Andres et al. (1999). The posterior part included the central, parvocellular, and superior parts of the medial division of the lateral habenular complex and the basal, magnocellular, marginal, oval, and parvocellular parts of the lateral division of the lateral habenular complex. To analyze the mediolateral differences in the LHb, all sections (−0.95 to −2.15 from the bregma) were divided into the medial and lateral parts using a line connecting the midpoints of the dorsal and ventral borders. The medial part included the anterior, central, marginal, parvocellular, and superior parts of the medial division of the lateral habenular complex and basal, magnocellular, marginal, and parvocellular parts of the lateral division of the lateral habenular complex. The lateral part included the anterior, central, and marginal parts of the medial division of the lateral habenular complex and basal, magnocellular, marginal, oval, and parvocellular parts of the lateral division of the lateral habenular complex. Positive cells were also counted in the basolateral amygdala, CA3 in the hippocampus, and region 30 in the cingulate cortex from three sections within the anterior −1.55 mm and posterior −2.15 mm ranges (Franklin and Paxinos, 2007). Each image was optically sectioned, and a series of confocal z-stack images were recorded at 2 μm intervals. The numbers of positive cells in all optical sections were counted using the ImageJ cell counter plug-in (National Institutes of Health; https://imagej.nih.gov/ij/plugins/cell-counter.html). Regarding multiple staining of HCR and IHC, we investigated the simultaneous detection of different mRNA expressions with HCR: *vglut1* and *vglut2*, *gad1* and *gad2*, *vgat* and *vglut3*, *gad1* and *vglut1*, *vglut2* and *gad2*, *vgat* and *pv*, *vglut2* and *gat*, *vglut2* (set1 and set2), *gad2* (set1 and set2) in the LHb, cingulate cortex, BLA, and hippocampus CA3. Different HCR and IHC signals in the images were obtained and analyzed separately. Each signal is represented as pseudocolors, namely, magenta, cyan, blue, and green. Combinations of pseudocolors were selected to increase signal visibility.

### Statistical analyses

Correlation analysis of *pv* genes with GABAergic and glutamatergic marker genes in single-cell RNA sequencing data was performed using R version 4.4.0 (R Core Team; https://www.*R*-projecrt.org/). Data are expressed as the mean ± standard error of the mean. The densities of PV neurons and percentages of neurotransmitter markers in PV neurons were statistically compared between the LHb and other brain regions using a one-way analysis of variance (ANOVA), followed by Tukey's honest significant difference (HSD) test. The percentage of *vglut2*-positive PV neurons was compared between the LHb and the cingulate cortex using Welch's *t* test. The percentages of neurotransmitter markers in PV neurons were compared between the medial and lateral LHb and between the anterior and posterior LHb using Welch's *t* test. Statistical significance was set for all tests, i.e., *p < 0.05, **p < 0.01, and ***p < 0.001. Analyses were performed using JMP Pro 14.2 (SAS Institute).

## Results

### Gene expression of glutamatergic and GABAergic markers in LHb *pv* neurons using transcriptome analysis

**Figure 1.**
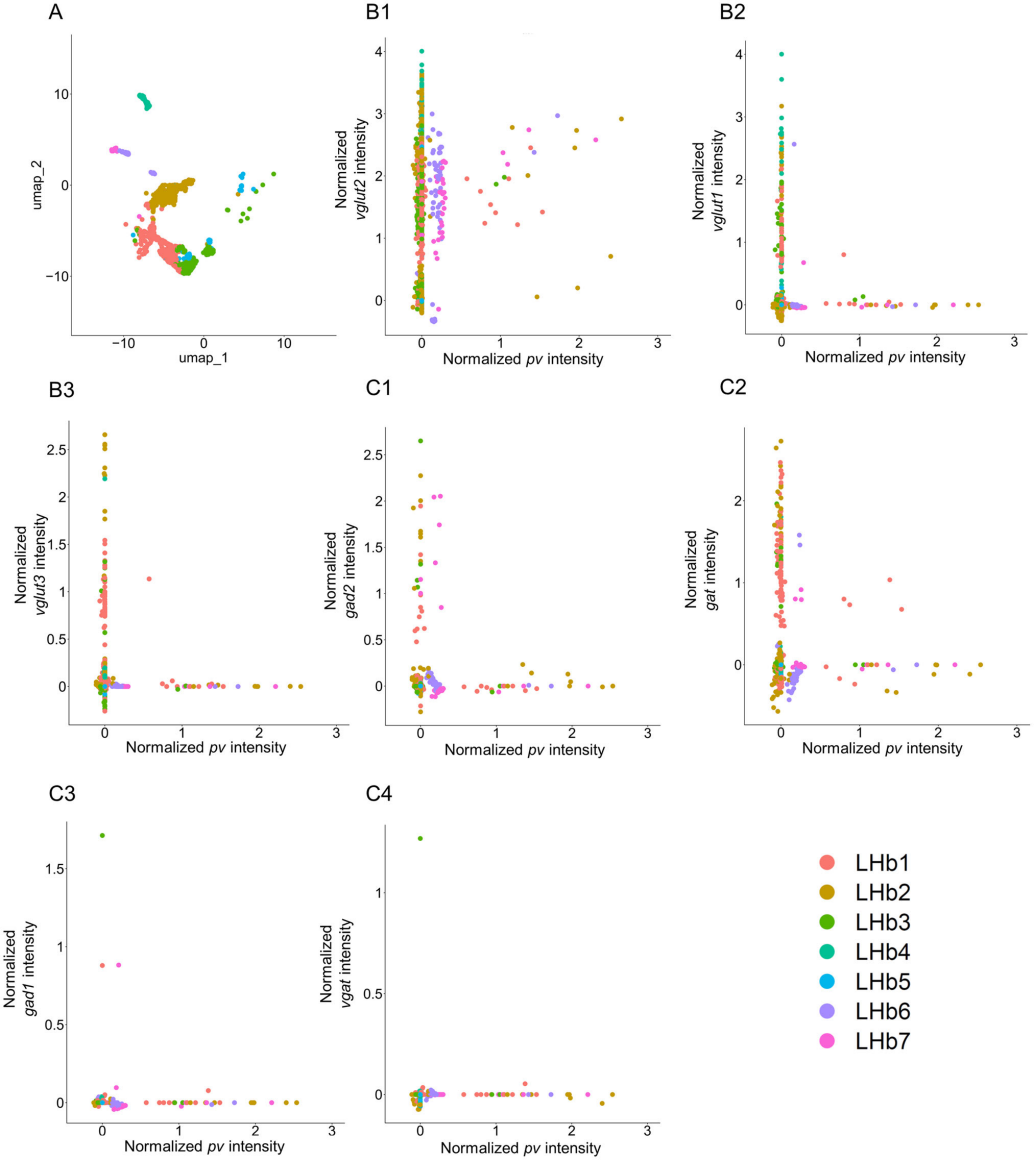
Gene expression of a glutamatergic marker in LHb *pv*-positive neurons. ***A***, The LHb neurons are classified into seven clusters by UMAP. The clusters are color coded as follows: LHb1 is red, LHb2 is yellow, LHb3 is yellowish green, LHb5 is light blue, LHb6 is purple, and LHb7 is pink. ***B***, Scatter plots indicate normalized expressions of *pv* and *vglut2*, *vglut1*, *vglut3*, *gad2*, *gat*, *gad1*, *vgat* on the *x*- and *y*-axes, respectively (*N *= 1,538). Plot colors indicate the clusters. LHb, lateral habenular nucleus. *pv*, *parvalbumin*; *gad1*, *glutamate decarboxylase 1*; *gad2*, *glutamate decarboxylase 2*; *vgat*, *vesicular GABA transporter*; *gat*, *GABA transporter*; *vglut1*, *vesicular glutamate transporter 1*; *vglut2*, *vesicular glutamate transporter 2*.

Seven LHb neuronal clusters were extracted from a previously published single-cell RNA sequencing dataset of adult mice by visualizing canonical gene markers in a Uniform Manifold Approximation and Projection (UMAP) space (Fig. 1*A*; Hashikawa et al., 2020). Gene expression levels of glutamatergic markers were examined in the neurons of the adult mouse LHb using the *pv* gene sequence (Fig. 1*B*1–3). Neurons expressing both *pv* and *vglut2* were frequently observed across the five clusters, and a significant positive correlation was observed (*r *= 0.068; *p *= 0.0080; Fig. 1*B*1). Conversely, only a few neurons expressing both *pv* and *vglut1* were observed, and no significant correlation was observed (Fig. 1*B*2; *r *= −0.027; *p *= 0.30). A few neurons expressing both *pv* and *vglut3* were observed, and there was no significant correlation (*r *= −0.026; *p *= 0.32; Fig. 1*B*3). The gene expression levels of GABAergic markers were examined in the neurons of the adult mouse LHb with the *pv* gene sequence (Fig. 1*C*1–4). Neurons that express the *pv* gene were observed to have minimal coexpression of the *gad2* and *gat* genes, with no significant correlation (Fig. 1*C*1,2; *gad2*: *r *= 0.013, *p *= 0.60; *gat*: *r *= −0.027, *p *= 0.30). Similarly, in the LHb neurons, there was almost no expression of *gad1* and *vgat*, and no correlation was observed (Fig. 1*C*3,4; *gad1*: *r *= −0.0040, *p *= 0.87; *vgat*: *r *= −0.0042, *p *= 0.87). Overall, in the LHb, a high percentage of *pv* neurons expressed the glutamatergic marker *vglut2*. In contrast, a low percentage of *pv* neurons expressed the GABAergic markers *gad2* and *gat*.

### Expression of glutamatergic markers in LHb PV neurons and other regions using HCR

**Figure 2.**
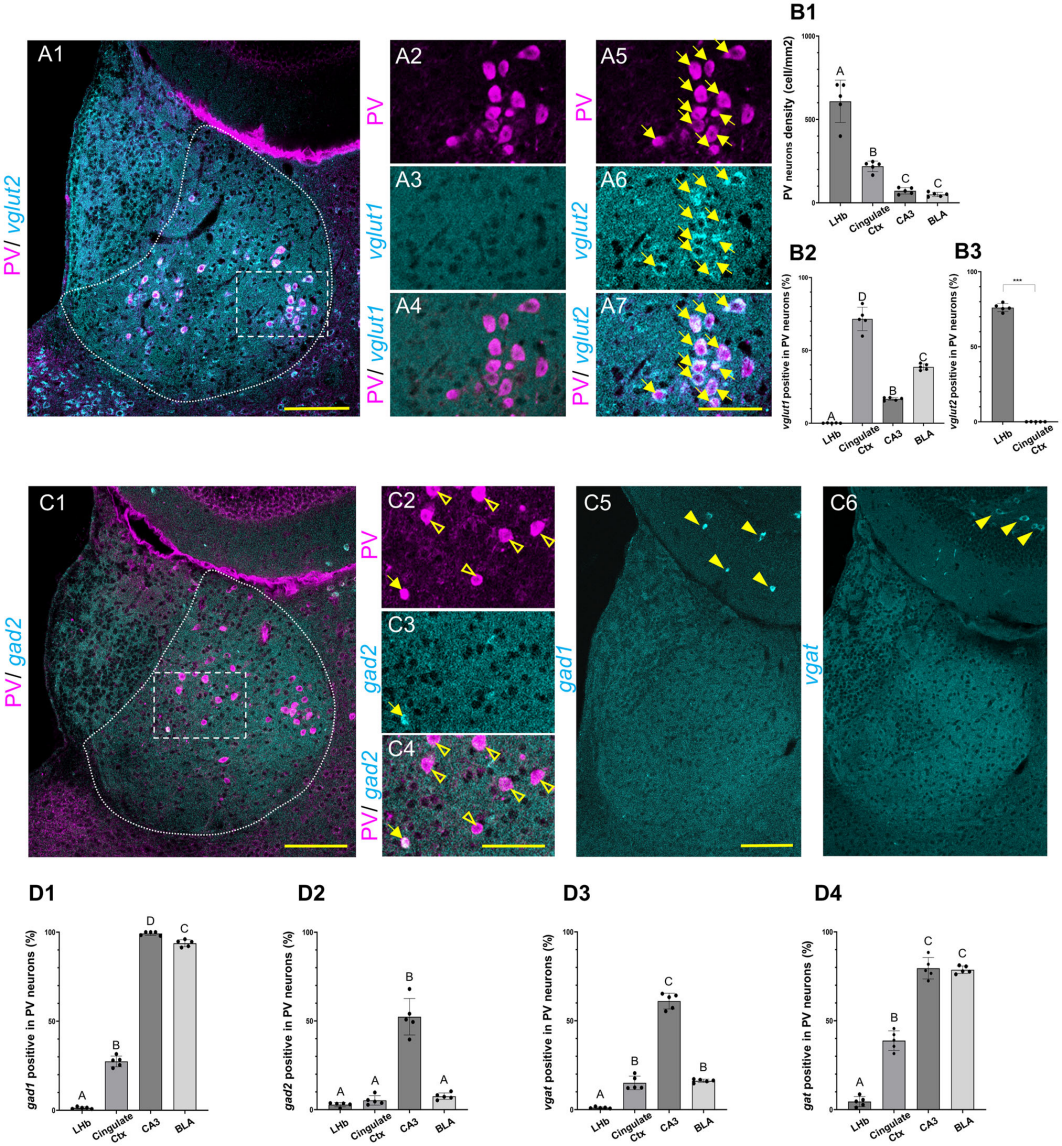
Heterogeneity of PV neurons in the expression of glutamatergic and GABAergic markers. ***A***, The expression of glutamatergic machinery in PV neurons in the LHb. *vglut2* (cyan) and PV (magenta) are double stained (***A*1**). ***A*5–7**, The dotted square area of ***A*1**. PV neurons (magenta in ***A*2**) and the expression of *vglut1* (cyan in ***A*3**) are shown (merged in ***A*4**). PV neurons (***A*5**) and the expression of *vglut2* (***A*6**) are shown (merged in ***A*7**). Arrows indicate double-positive for PV and *vglut2*. ***B***, The expression of glutamatergic machinery in PV neurons in the LHb and other brain regions. Densities of PV neurons are compared between the LHb and other brain regions (***B*1**). Percentages of *vglut1*- and *vglut2*-positive (***B*2** and ***B*3**, respectively) in PV neurons are compared between the LHb and other brain regions. ***C***, The expression of GABAergic machinery in PV neurons in the LHb. *gad2* (cyan) and PV (magenta) cells are double stained with HCR and IHC, respectively (***C*1**). ***C*2–4**, The dotted square area of ***C*1**. PV neurons (***C*2**) and the expression of *gad2* (***C*3**) are shown (merged in ***C*4**). Open arrowheads indicate PV neurons. Arrows indicate double positives for PV and *gad2*. Expressions of *gad1* (***C*5**) and *vgat* (***C*6**) are shown; closed arrowheads indicate the positive neurons in the hippocampus, but not in the LHb. ***D***, Percentages of the GABAergic machinery expression are compared between the LHb and other brain regions. Percentages of *gad1*-(***D*1**), *gad2*-(***D*2**), *vgat*-(***D*3**), and *gat*-positive PV neurons (***D*4**) are shown. ***C*5**, ***C*6**, Z-stack composite images with 10 confocal serial optical sections, whereas the others are single-optical sections of confocal images. Scale bars: 100 µm (***A*1**, ***C*1**, ***C*5,6**), 50 µm (***A*2–7**, ***C*2–4**). LHb, lateral habenular nucleus; cingulate Ctx, cingulate cortex; CA3, CA3 region of the hippocampus; BLA, basolateral amygdala; PV, parvalbumin; *gad1*, *glutamate decarboxylase 1*; *gad2*, *glutamate decarboxylase 2*; *vgat*, *vesicular GABA transporter*; *gat*, *GABA transporter*; *vglut1*, *vesicular glutamate transporter 1*; *vglut2*, *vesicular glutamate transporter 2*. *N* = 5 mice for each group. “A,” “B,” “C,” and “D” are significantly different (Tukey HSD test). For ***B*1**, *p* < 0.001 “A,” “B,” “A”–“C,” *p* < 0.05 “B,” “C.” For ***B*2**, *p* < 0.001 “A,” “B,” “A”–“C,” “A”–“D,” “B,” “C,” “B”–“D,” and “C,” “D.” For ***B*3**, ***p < 0.001. Welch's *t* test. For ***D*1–4**, *p* < 0.001 “A,” “B,” “A”–“C,” “A”–“D,” “B,” “C,” and “B”–“D.” *p* < 0.05 “C,” “D.”

The expression of glutamatergic markers was further examined in the LHb (Fig. 2*A*1–7). PV neurons were found to be distributed in both the medial and lateral parts of the LHb (Fig. 2*A*1), almost none of which were positive for *vglut1* (Fig. 2*A*2–4); however, many PV neurons were positive for *vglut2* (Fig. 2*A*1,5–7). In contrast, most PV neurons in the cingulate cortex were positive for *vglut1*; however, almost none were positive for *vglut2* (Fig. 2-1*A*1–7). Moreover, PV neurons in the hippocampus CA3 and basolateral amygdala were positive for *vglut1*; however, no PV neurons were positive for *vglut2* (Fig. 2-1*B*1–7,*C*1–7). In addition, positivity for *vglut3* was rarely observed in PV neurons in the LHb or other regions (data not shown).

10.1523/ENEURO.0069-24.2024.f2-1 Figure 2-1 Heterogeneity of PV neurons in the expression of glutamatergic markers in the cingulate cortex, the hippocampus CA3, and the basolateral amygdala. **A** The expression of glutamatergic machinery in PV neurons in the cingulate cortex. *vglut1* (cyan) and PV (magenta) are double-stained with HCR and IHC, respectively (**A1**). **A2-7** shows the dotted square area of **A1**. PV neurons (magenta in **A2**) and expression of *vglut1* (cyan in **A3**) are shown (merged in **A4**). PV neurons (magenta in **A5**) and expression of *vglut2* (cyan in **A6**) are shown (merged in **A7**). Arrows indicate double-positive for PV and *vglut1*. **B** The expression of glutamatergic machinery in PV neurons in the hippocampus (CA3 area). *vglut1* (cyan) and PV (magenta) are double-stained with HCR and IHC, respectively (**B1**). **B2-7** shows the dotted square area of **B1**. PV neurons (magenta in **B2**) and expression of *vglut1* (cyan in **B3**) are shown (merged in **B4**). PV neurons (magenta in **B5**) and expression of *vglut2* (cyan in **B6**) are shown (merged in **B7**). Arrows indicate double-positive for PV and *vglut1*. **C** Expression of glutamatergic machinery in PV neurons in the basolateral amygdala. *vglut1* (cyan) and PV (magenta) are double-stained with HCR and IHC, respectively (**C1**). **C2-7** shows the dotted square area of **C1**. PV neurons (magenta in **C2**) and expression of *vglut1* (cyan in **C3**) are shown (merged in **C4**). PV neurons (magenta in **C5**) and expression of *vglut2* (cyan in **C6**) are shown (merged in **C7**). Arrows indicate double-positive for PV and *vglut1*. All images are single optical sections of confocal images. Scale bars: 100 µm (**A1**, **B1**, **C1**), 50 µm (**A2-7**, **B2-7**, **C2-7**). PV, parvalbumin. *vglut1*, *vesicular glutamate transporter 1*. *vglut2*, *vesicular glutamate transporter 2*. Download Figure 2-1, TIF file.

10.1523/ENEURO.0069-24.2024.f2-2 Figure 2-2 Heterogeneity of PV neurons in the expression of GABAergic markers in the cingulate cortex, hippocampus CA3, and basolateral amygdala. **A** The expression of GABAergic machinery in PV neurons in the cingulate cortex. *gad1* (cyan) and PV (magenta) are double-stained with HCR and IHC, respectively (**A1**). **A2-4** shows the dotted square area of **A1**. PV neurons (magenta in **A2**) and expression of *gad1* (cyan in **A3**) are shown (merged in **A4**). PV neurons (magenta in **A5**) and expression of *gad2* (cyan in **A6**) are shown (merged in **A7**). PV neurons (magenta in **A8**) and expression of *vgat* (cyan in **A9**) are shown (merged in **A10**). Open arrowheads indicate PV neurons. Arrows indicate positive for *gad1*, *gad2*, or *vgat* in PV neurons. Closed arrowheads indicate *vgat* positive neurons. **B** The expression of GABAergic machinery in PV neurons in the hippocampus (CA3 area). *gad1* (cyan) and PV (magenta) are double-stained with HCR and IHC, respectively (**B1**). **B2-4** shows the dotted square area of **B1**. PV neurons (magenta in **B2**) and expression of *gad1* (cyan in **B3**) are shown (merged in **B4**). PV neurons (magenta in **B5**) and expression of *gad2* (cyan in **B6**) are shown (merged in **B7**). PV neurons (magenta in **B8**) and expression of *vgat* (cyan in **B9**) are shown (merged in **B10**). Open arrowheads indicate PV neurons. Arrows indicate positive for *gad1*, *gad2*, or *vgat* in PV neurons. Closed arrowheads indicate *gad1* or *gad2* positive neurons. **C** The expression of GABAergic machinery in PV neurons in the basolateral amygdala. *gad1* (cyan) and PV (magenta) are double-stained with HCR and IHC, respectively (**C1**). **C2-4** shows the dotted square area of **C1**. PV neurons (magenta in **C2**) and expression of *gad1* (cyan in **C3**) are shown (merged in **C4**). PV neurons (magenta in **C5**) and expression of *gad2* (cyan in **C6**) are shown (merged in **C7**). PV neurons (magenta in **C8**) and expression of *vgat* (cyan in **C9**) are shown (merged in **C10**). Open arrowheads indicate PV neurons. Arrows indicate positive for *gad1* or *vgat* in PV neurons. All images are single optical sections of confocal images. Scale bars: 100 µm (**A1**, **B1**, **C1**), 50 µm (**A2-10**, **B2-10**, **C2-10**). PV, parvalbumin. *gad1*, *glutamate decarboxylase 1*. *gad2*, *glutamate decarboxylase 2*. *vgat*, *vesicular GABA transporter*. Download Figure 2-2, TIF file.

**Figure 3.**
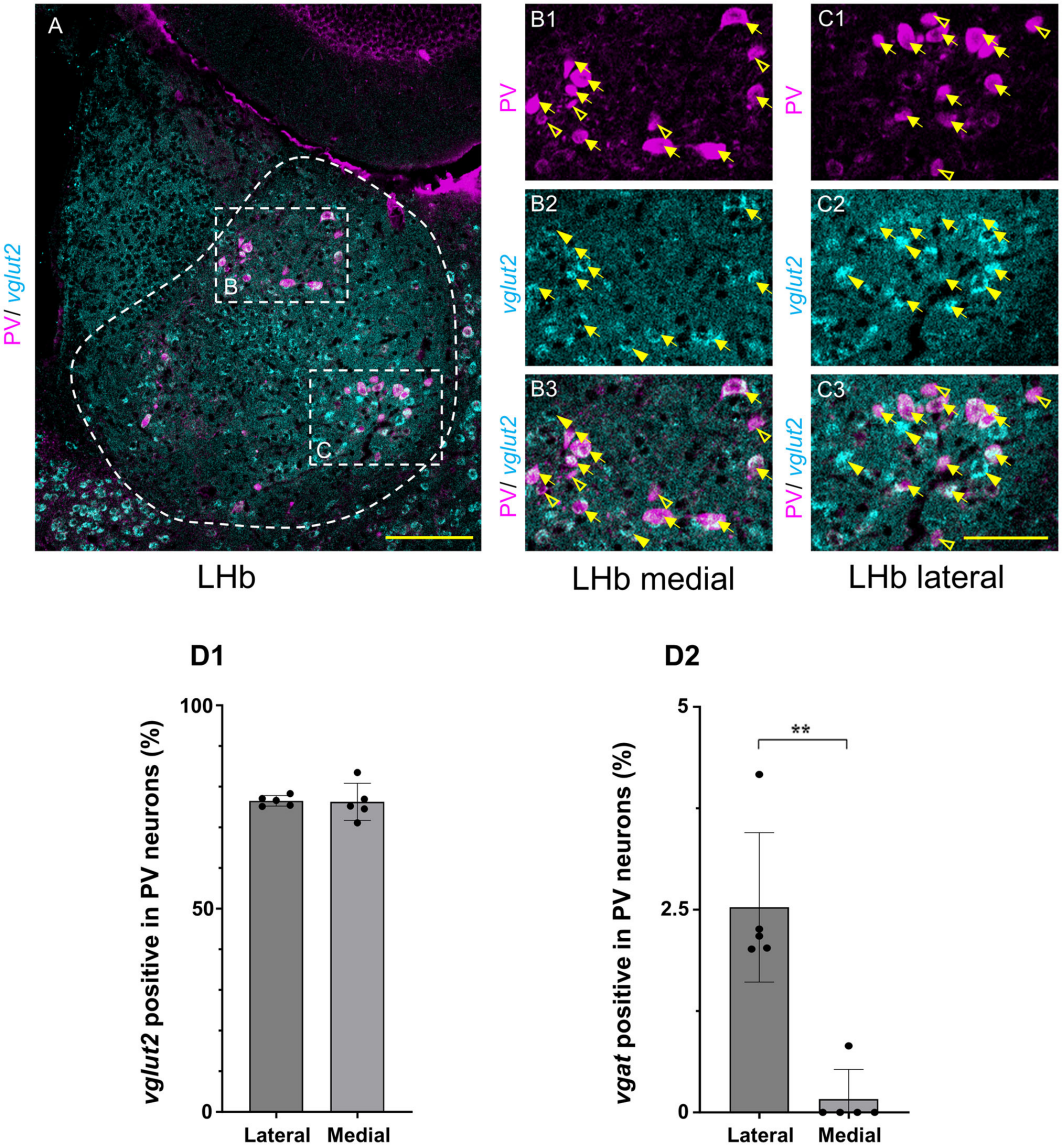
Mediolateral distribution of glutamatergic and GABAergic PV neurons in the LHb. ***A***, The expression of *vglut2* (cyan) in the LHb PV neurons (magenta). PV and *vglut2* are detected using IHC and HCR, respectively. The dotted square areas ***B*** and ***C*** are shown in ***B*1–3** and ***C*1–3**, respectively. ***B***, In the medial LHb PV neurons (magenta in ***B*1**), the expression of *vglut2* (cyan in ***B*2**) is shown (merged in ***B*3**). ***C***, In the lateral LHb PV neurons (magenta in ***C*1**), the expression of *vglut2* (cyan in ***C*2**) is shown (merged in ***C*3**). Open and closed arrowheads indicate PV- and *vglut2*-positive neurons, respectively. Arrows indicate double-positive for PV and *vglut2*. All images are single-optical sections. Scale bars: 100 µm (***A***), 50 µm (***B*1–3**, ***C*1–3**). ***D***, Percentages of *vglut2*-(***D*1**) and *vgat*-positive PV neurons (***D*2**) are compared between the medial and lateral LHb. *N* = 5. **p < 0.01. Welch's *t* test. LHb, lateral habenular nucleus; PV, parvalbumin; *vglut2*, *vesicular glutamate transporter 2*; *vgat*, *vesicular GABA transporter*.

10.1523/ENEURO.0069-24.2024.f2-3 Figure 2-3 A CSV file of extended data table supporting Figure 2. Download Figure 2-3, XLSX file.

The densities of PV neurons in the LHb, cingulate cortex, hippocampus CA3, and basolateral amygdala differed significantly (*F*_3,16_ = 76.28, *p *< 0.0001, one-way ANOVA), with the density of the neurons in the LHb (608.73 ± 56.66 cells/mm^2^, mean ± standard error) being significantly higher in comparison with the densities of the cingulate cortex, hippocampus CA3, and basolateral amygdala (218.86 ± 14.13, 71.53 ± 8.41, and 49.59 ± 5.73 cells/mm^2^, respectively, *p *< 0.0001, Tukey HSD test; Fig. 2*B*1). The difference in the density of PV neurons in the LHb between this study and that of Nakamura et al. (2021) is thought to arise from the variation in the concentration of the primary antibody, difference in staining methods, and use of microscopes with different precisions. Percentages of *vglut1*-positive PV neurons differed significantly between the regions (*F*_3,16_ = 272.64; *p *< 0.0001), with the percentage in the LHb (0.104 ± 0.064%) being significantly lower than that in the other regions (71.57 ± 3.59, 16.64 ± 0.47, and 38.61 ± 0.94% for the cingulate cortex, hippocampus CA3, and basolateral amygdala, respectively. All *p *< 0.0001; Fig. 2*B*2). The percentage of *vglut2*-positive PV neurons was significantly higher in the LHb than in the cingulate cortex (76.08 ± 1.20% in the LHb and 0.016 ± 0.016% in the cingulate cortex, *p *< 0.0001, Welch's *t* test; Fig. 2*B*3). The percentages of *vglut3*-positive PV neurons were 0.047 ± 0.047% in the LHb, 0.074 ± 0.037% in the cingulate cortex, 0.26 ± 0.16% in the hippocampus CA3, and 0% in the basolateral amygdala (figures not shown). Overall, a high percentage of PV neurons in the LHb express the glutamatergic marker *vglut2*.

### Expression of GABAergic markers in LHb PV neurons and other regions using HCR

Although most neurons were negative, one was positive for *gad2* (Fig. 2*C*1–4). Almost none of the cells were positive for *gad1* and *vgat* expression. In contrast, *gad1-* and *vgat-*positive cells were detected in the hippocampus (Fig. 2*C*5,6). PV neurons were predominantly distributed in Layers II–III and V of the cingulate cortex and were positive for *gad1*, *gad2*, and *vgat* (Fig. 2-2*A*1–10). PV neurons were scattered in the CA3 region of the hippocampus, and several were positive for *gad1*, *gad2*, and *vgat* (Fig. 2-2*B*1–10). A small number of PV neurons are found in the basolateral amygdala. Several PV neurons were positive for *gad1* and *vgat*; however, only a few were positive for *gad2* (Fig. 2-2*C*1–10).

Percentages of *gad1*-positive PV neurons differed significantly between the regions (*F*_3,16_ = 3,229.71, *p *< 0.0001, one-way ANOVA), with the percentage of *gad1*-positive PV neurons in the LHb (1.42 ± 0.33%) being significantly lower compared with other regions (cingulate cortex, hippocampus CA3, and basolateral amygdala; 27.43 ± 1.35, 99.24 ± 0.47, and 93.76 ± 0.88%, respectively, *p *< 0.0001; Fig. 2*D*1). The percentage of *gad2*-positive PV neurons was significantly different between the regions (*F*_3,16_ = 94.78; *p *< 0.0001). The percentage of *gad2*-positive PV neurons was significantly lower in the LHb than that in the hippocampus CA3 (2.93 ± 0.57 vs 52.36 ± 4.60%, *p *< 0.0001). However, it was not significantly different from the percentages of *gad2*-positive neurons in the cingulate cortex and the basolateral amygdala (5.24 ± 1.19%, *p *= 0.906 and 7.55 ± 0.84%, *p *= 0.550, respectively; Fig. 2*D*2). Percentages of *vgat*-positive PV neurons were significantly different between the regions (*F*_3,16_ = 381.46; *p *< 0.0001), with the percentage in the LHb (1.14 ± 0.22%) being significantly lower than that in the other regions (15.03 ± 1.71, 61.06 ± 1.99, and 16.21 ± 0.42% for the cingulate cortex, hippocampus CA3, and basolateral amygdala, respectively. All *p *< 0.0001; Fig. 2*D*3). The percentage of *gat*-positive PV neurons was also significantly different between the regions (*F*_3,16_ = 328.53; *p *< 0.0001). The percentage of *gat*-positive PV neurons in the LHb (4.52 ± 1.27%) was significantly lower than that in the other regions (38.81 ± 2.46, 79.53 ± 2.68, and 78.68 ± 0.96% for the cingulate cortex, hippocampus CA3, and basolateral amygdala, respectively. All *p *< 0.0001; Fig. 2*D*4). Therefore, a lower percentage of PV neurons expressed GABAergic markers in the LHb than in other regions.

### Mediolateral distribution of glutamatergic and GABAergic PV neurons in the LHb

The expression of *vglut2* in the PV neurons was compared between the medial and lateral LHb. The majority of PV neurons were positive for *vglut2* in both the medial (Fig. 3*A*,*B*1–3) and lateral LHb (Fig. 3*A*,*C*1–3) regions. The percentages of *vglut2* positive PV neurons did not differ significantly between the medial and lateral LHb (76.25 ± 2.04 vs 76.5 ± 0.57%, *p *= 0.912, Welch's *t* test; Fig. 3*D*1).

The expression of the GABAergic marker *vgat* in PV neurons was also compared between the medial and lateral LHb. The percentage of *vgat*-positive PV neurons was significantly higher in the lateral LHb than in the medial LHb (*vgat*: 2.53 ± 0.41 vs 0.16 ± 0.16%, *p *= 0.007, Welch's *t* test; Fig. 3*D*2). We further examined the expression of *vgat* in *pv*-mRNA-positive neurons using HCR. The *pv*-mRNA-positive neurons comprised 29.71 ± 0.32% of the PV (protein)-positive neurons in the LHb. Among the *pv*-mRNA-positive neurons, 8.37 ± 1.19% were *vgat* positive. The percentage of *vgat*-positive *pv-*mRNA-positive neurons was significantly higher in the lateral LHb than the medial LHb (22.03 ± 3.53 vs 0 ± 0%, *p *= 0.025, Welch's *t* test; Fig. 3-1). Therefore, the medial and lateral LHb differed topographically in *vgat*, but not in the expression of *vglut2*.

10.1523/ENEURO.0069-24.2024.f3-1 Figure 3-1 Mediolateral distribution of GABAergic *pv* mRNA positive neurons in the LHb. **A** PV-protein (magenta), *pv*-mRNA(green), and *vgat* (cyan) are triple-stained with IHC and HCR in the LHb (**A1**). The dotted square area of **A1** is shown in **A2**-**5**. In PV-protein positive neurons (magenta in **A2**), *pv*-mRNA positive neurons (green in **A3**), and expression of *vgat* (cyan in **A4**) is shown (merged in **A5**). Open arrowheads indicate PV-protein positive neurons. Arrows indicate triple-positive for PV-protein, *pv-*mRNA, and *vgat*. Closed arrowheads indicate *pv-*mRNA positive neurons. **B** Percentages of *vgat* expression in the *pv*-mRNA positive neurons are compared between the medial and lateral LHb (**B1**). The *pv*-mRNA positive neurons are counted, and the *vgat* positive neurons among them are counted in the medial (**B2**) and lateral LHb (**B3**). The percentages of *vgat* positive neurons in the *pv*-mRNA positive neurons were significantly higher in the lateral LHb than those in the medial (*vgat*: 22.03 ± 3.53% in lateral and 0 ± 0% in medial, *p* = 0.02, Welch's *t*-test, HCR, **B1**). In the medial LHb, 102 *pv*-mRNA positive neurons were observed; among them, no neurons expressed *vgat* (**B2**). In contrast, 63 *pv*-mRNA positive neurons were observed; among them, the 14 neurons expressed *vgat* in the lateral LHb (**B3**). All images are single optical sections. Scale bars: 100 µm (**A1**), 50 µm (**A2-5**). LHb, lateral habenular nucleus. PV, parvalbumin (protein). *pv, parvalbumin* (mRNA)*. vgat*, *vesicular GABA transporter. N* = 3 mice. *p < 0.05. Welch's *t*-test. Download Figure 3-1, TIF file.

10.1523/ENEURO.0069-24.2024.f3-2 Figure 3-2 A CSV file of extended data table supporting Figure 3. Download Figure 3-2, XLSX file.

### Anteroposterior distribution of glutamatergic and GABAergic PV neurons in the LHb

**Figure 4.**
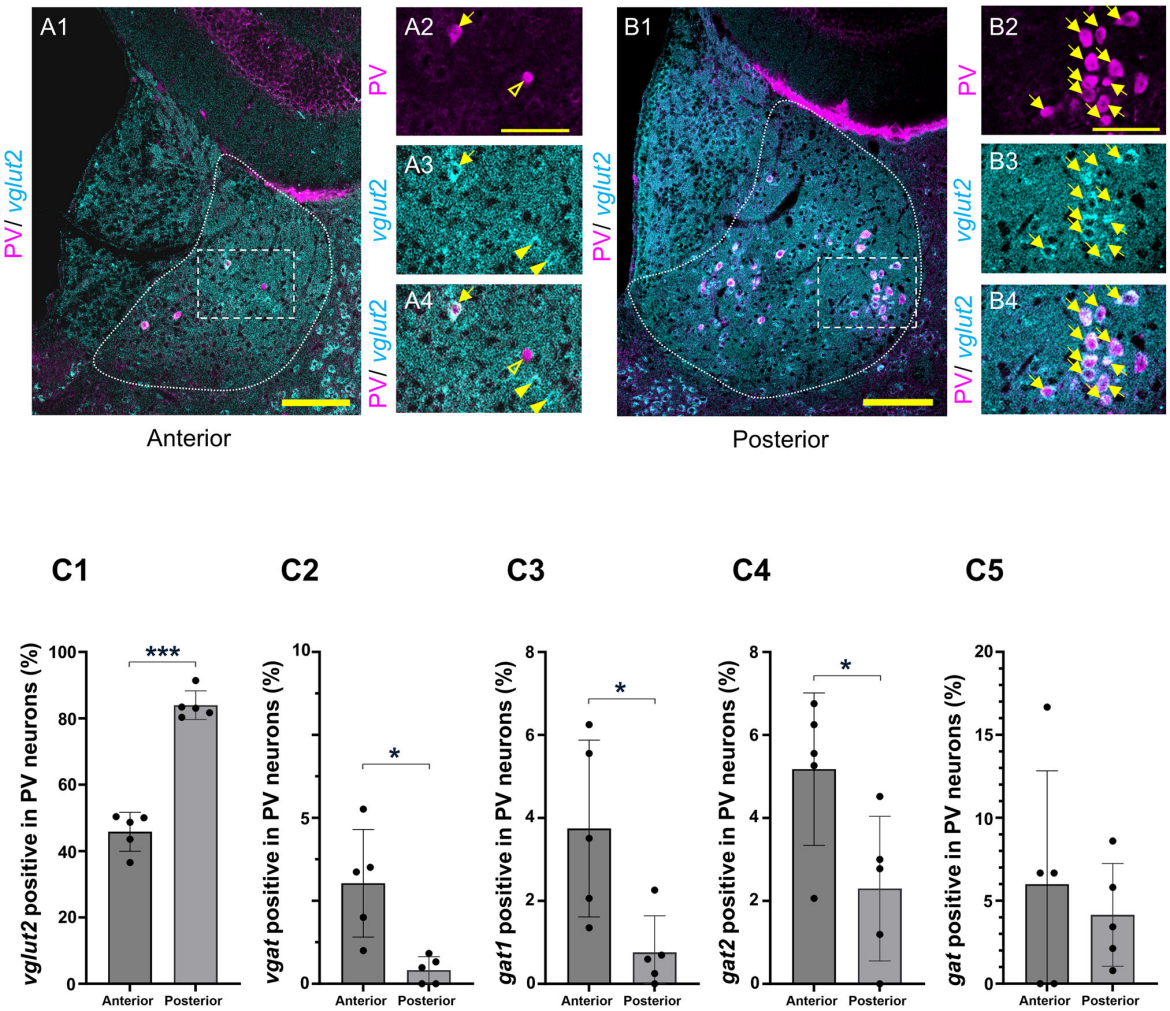
Anteroposterior distribution of glutamatergic and GABAergic PV neurons in the LHb. ***A***, The expression of *vglut2* (cyan) in the anterior LHb PV neurons (magenta; ***A*1**). PV and *vglut2* are detected using IHC and HCR, respectively. The dotted square area of ***A*1** is shown in ***A*2–4**. In PV neurons (magenta in ***A*2**), the expression of *vglut2* (cyan in ***A*3**) is shown (merged in ***A*4**). ***B***, The expression of *vglut2* (cyan) in the posterior LHb PV neurons (magenta; ***B*1**). The dotted square area of ***B*1** is shown in ***B*2–4**. In the PV neurons (magenta in ***B*2**), the expression of *vglut2* (cyan in ***B*3**) is shown (merged in ***B*4**). Open and closed arrowheads indicate PV- and *vglut2*-positive neurons, respectively. Arrows indicate double positive for PV and *vglut2*. All images are single-optical sections. Scale bars: 100 µm (***A*1**, ***B*1**), 50 µm (***A*2–4**, ***B*2–4**). ***C***, Percentages of glutamatergic and GABAergic machinery expression are compared between the anterior and posterior LHb. Percentages of *vglut2-*(***C*1**), *vgat-*(***C*2**), *gad1*-(***C*3**), *gad2*-(***C*4**), and *gat*-positive PV neurons (***C*5**) are compared between the anterior and posterior LHb. *N* = 5. *p < 0.05; ***p < 0.001. Welch's *t* test. LHb, lateral habenular nucleus; PV, parvalbumin; *vglut2*, *vesicular glutamate transporter 2*; *vgat*, *vesicular GABA transporter*.

The expression of *vglut2* in the PV neurons was compared between the anterior and posterior LHb. Only a few PV neurons in the anterior LHb expressed *vglut2* (Fig. 4*A*1–4), whereas many in the posterior LHb expressed this gene (Fig. 4*B*1–4). The percentage of *vglut2*-positive PV neurons was significantly lower in the anterior LHb than in the posterior LHb (45.83 ± 2.62% in the anterior, 83.97 ± 1.93% in the posterior, *p *< 0.0001, Welch's *t* test; Fig. 4*C*1).

10.1523/ENEURO.0069-24.2024.f4-1 Figure 4-1 A CSV file of extended data table supporting Figure 4. Download Figure 4-1, XLSX file.

We further compared the expression of GABAergic markers in the PV neurons between the anterior and posterior LHb. Although a small number of PV neurons expressed the GABAergic markers in the LHb, the percentage of *vgat* positive PV neurons was significantly higher in the anterior LHb than in the posterior LHb (*vgat*: 3.02 ± 0.73 vs 0.32 ± 0.197%, *p *= 0.018, Welch's *t* test, Fig. 4*C*2). The percentages of *gad1*- and *gad2*-positive PV neurons in the anterior LHb were significantly higher than those in the posterior (*gad1*: 3.75 ± 0.95% in anterior and 0.76 ± 0.39% in posterior, *p *= 0.03, Welch's *t* test; Fig. 4*C*3; *gad2*: 5.18 ± 0.82% in anterior and 2.30 ± 0.78% in posterior, *p *= 0.03; Fig. 4*C*4). The expressions of *gat*-positive PV neurons were not significantly different between the anterior and posterior LHb (6.0 ± 3.06 vs 4.15 ± 1.39%, *p *= 0.6; Fig. 4*C*5). The anterior and posterior LHb showed significant differences in the expression of *vglut2*, *vgat*, *gad1*, and *gad2* but not in the expression of *gat*.

### Coexpression of glutamatergic and GABAergic markers in LHb PV neurons

**Figure 5.**
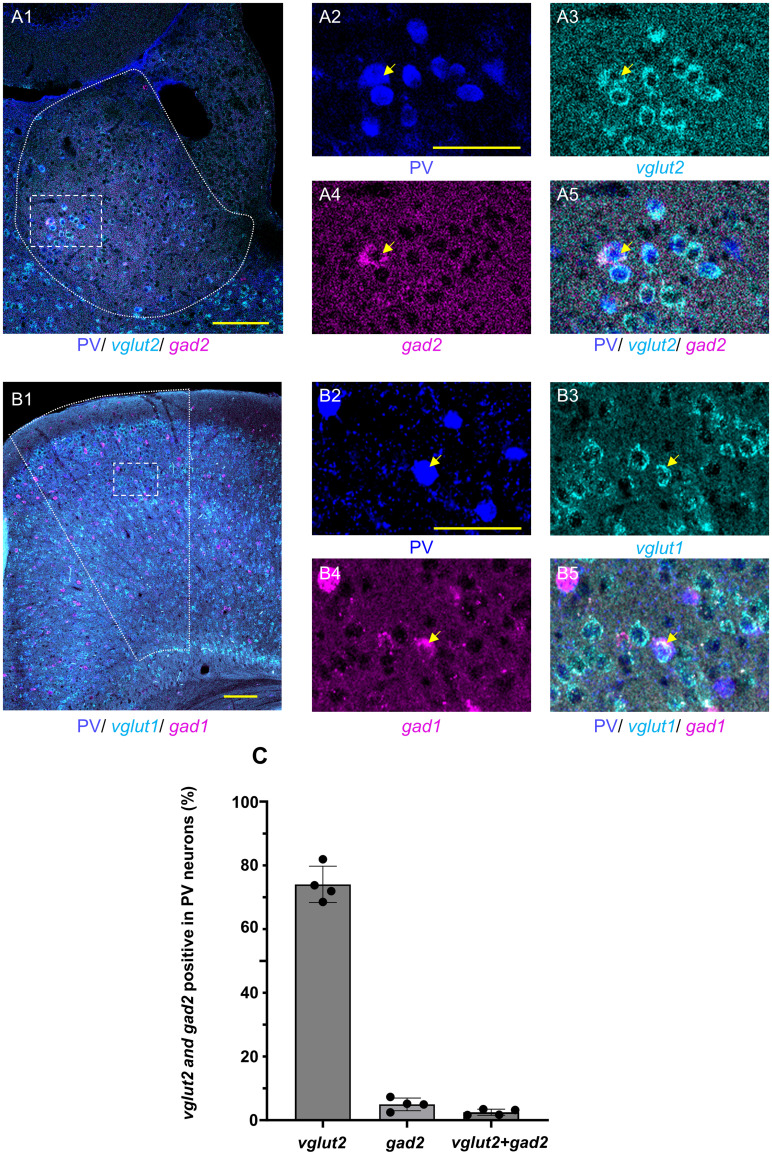
Coexpression of glutamatergic and GABAergic machinery in the PV neurons. ***A***, PV (blue), *vglut2* (cyan), and *gad2* (magenta) are triple stained in the LHb using IHC and HCR (***A*1**). The dotted square area of ***A*1** is shown in ***A*2–5**. In PV neurons (blue in ***A*2**), the expression of *vglut2* (cyan in ***A*3**) and *gad2* (magenta in ***A*4**) is shown (merged in ***A*5**). Arrows indicate triple positive for PV, *vglut2*, and *gad2*. ***B***, PV (blue), *vglut1* (cyan), and *gad1* (magenta) are triple stained in the cingulate cortex using IHC and HCR (***B*1**). The dotted square area of ***B*1** is shown in ***B*2–5**. In PV neurons (blue in ***B*2**), the expression of *vglut1* (cyan in ***B*3**) and *gad1* (magenta in ***B*4**) is shown (merged in ***B*5**). Arrows indicate triple positive for PV, *vglut1*, and *gad1*. All images are single-optical sections. Scale bars: 100 µm, (***A*1**, ***B*1**). 50 µm, (***A*2–5**, ***B*2–5**). ***C***, Percentage of *vglut2*, *gad2*, and double-positive PV neurons in the LHb. *N* = 4. LHb, lateral habenula; PV, parvalbumin; *vglut1*, *vesicular glutamate transporter 1*; *vglut2*, *vesicular glutamate transporter 2*; *gad1*, *glutamate decarboxylase 1*; *gad2*, *glutamate decarboxylase 2*.

PV neurons that express both glutamatergic and GABAergic markers have been identified in the LHb. In this study, we found that some PV neurons in the LHb exhibited double positivity for *vglut2* and *gad2* (Fig. 5*A*1–5). A total of 2.51 ± 0.48% of the PV neurons were double positive for *vglut2* and *gad2* (Fig. 5*C*). Conversely, 54.38 ± 15.08% of the double-positive cells expressed the PV protein.

10.1523/ENEURO.0069-24.2024.f5-1 Figure 5-1 A CSV file of extended data table supporting Figure 5. Download Figure 5-1, XLSX file.

In contrast, double-positive cells for *vglut1* and *gad1* were observed in the cingulate cortex (Fig. 5*B*1–5), with 6.72 ± 1.18% of the PV neurons exhibiting double positivity. Other cell-type markers, including *serotonin transporter*, *choline acetyltransferase*, and *tyrosine hydroxylase* mRNA, were not observed in PV neurons in the LHb (data not shown).

## Discussion

This study aimed to deepen our understanding of the LHb by collecting information regarding the heterogeneity of LHb PV neurons through the comprehensive investigation of the expression of cell-type markers using transcriptome analysis, HCR, and IHC. The analysis of previously published single-cell RNA sequencing data (Hashikawa et al., 2020) confirmed that many PV gene-positive neurons were *vglut2* gene-positive neurons (Fig. 1*B*1) as reported by Webster et al. (2020). Furthermore, this study quantitatively elucidated and specified the percentages of *vglut1*-, *vglut2*-, and *vglut3*-positive PV neurons along with *gad1*-, *gad2*-, *vgat*-, and *gat*-positive PV neurons. We demonstrated that a large percentage of the PV neurons were glutamatergic in the LHb, with 76.08 ± 1.20% of them showing positivity for *vglut2* (Fig. 2*B*2). The majority of PV neurons are glutamatergic, meaning that they release glutamate from *vglut2*-positive vesicles. In contrast, the percentage of GABAergic neurons was lower, with only 1.42 ± 0.33% of the PV neurons showing positivity for *gad1*, 2.93 ± 0.57% for *gad2*, 1.14 ± 0.22% for *vgat*, and 4.52 ± 1.27% for *gat* (Fig. 2*D*1–4). Previous studies on PV neurons in the LHb have reported the following positive percentages: 0% for GAD1 in GFP knock-in mice, 4.8% for GAD2 in mCherry mice (Nakamura et al., 2021), and 56.4% (Nakamura et al., 2021) and 8.8% (Webster et al., 2020) for GABA immunoreactivity, using different anti-GABA antibodies and protocols. The reasons underlying the low gene expression of the GABAergic transmission machinery in contrast to GABA immunoreactivity remains a question for future research.

There are several brain regions that contain a high proportion of glutamatergically positive PV neurons, along with the LHb, *vglut2* in the LHb, and *vglut1* in other areas. Herein, we directly demonstrated the presence of vesicular glutamate transporter mRNA in PV neurons, in contrast to Xu et al. (2022), who observed all cells that expressed PV and *vglut2* proteins simultaneously at least once by using the cre and FlpO systems in many brain regions, including the LHb. PV neurons in the cingulate cortex expressed *vglut1* at a percentage comparable to that of LHb neurons (Fig. 2*B*1,2); however, they expressed GABAergic markers at a higher percentage than LHb neurons (Fig. 2*D*1–4). In contrast, PV neurons in the hippocampus CA3 and basolateral amygdala expressed GABAergic markers (Fig. 2*D*1–4) at high percentages and *vglut1* in moderate percentages (Fig. 2*B*1). The percentage of *gad1* in amygdala PV neurons was consistent with a previous in situ study using rats (Yamaguchi et al., 2021). Furthermore, our results showed that the proportions of glutamatergic and GABAergic PV neurons differed depending on the brain region. A large percentage of the PV neurons in the LHb and cingulate cortex were glutamatergic, expressing *vglut2* and *vglut1*, respectively. In contrast, a large percentage of the PV neurons in the hippocampus CA3 and basolateral amygdala were GABAergic. Therefore, it is thought that PV neurons play different roles in each brain region, either excitatory or inhibitory, indicating that future studies should not rule out PV as a GABAergic marker without confirmation in each brain region. These results not only support previous findings that PV neurons are heterogeneous in terms of the expression of transmitter machinery but also clarify the quantitative composition of the heterogeneity, i.e., the proportion of PV neurons.

In addition, we demonstrated that PV neurons coexpressed *vglut2* and *gad2* in the LHb, as well as *vglut1* and *gad1* in the cingulate cortex (Fig. 5*A,B*). The presence of *vglut2* and *gad2* double-positive neurons has also been reported in the LHb (Quina et al., 2020). Xu et al. (2022) further reported 19 regions, excluding the LHb-contained neurons coexpressing *vglut2* and *vgat* by using in situ hybridization. These reports did not elucidate the percentage of the PV neurons. However, in the present study, we revealed that 54.38 ± 15.08% of the double-positive neurons in the LHb were PV positive (Fig. 5*C*). These findings demonstrate the heterogeneity of PV neurons, confirming that there are three different subsets of PV neurons: glutamatergic, GABAergic, and double-positive for glutamatergic and GABAergic machinery.

By comparing the percentages of the subsets, this study clarified the topographic organization of the LHb. We further uncovered the anteroposterior topography, which has not been previously reported. The percentage of *vglut2*-positive PV neurons was significantly higher in the posterior LHb (Fig. 4*C*1). In contrast, the percentage of GABAergic PV neurons positive for *gad1*, *gad2*, and *vgat* was significantly higher in the anterior LHb (Fig. 4*C*2–4), although GABAergic PV neurons were a minority in the LHb. Therefore, LHb was topographically organized anteroposteriorly in both glutamatergic and GABAergic PV neurons. These results suggest a possible link between the anteroposterior topography of PV neurons and the subnuclear topographic organization (Andres et al., 1999; Geisler et al., 2003; Aizawa et al., 2012), as well as with the functional topography following stress activation (Ichijo et al., 2015). In addition, we confirmed that the GABAergic neurons in the LHb were topographically organized mediolaterally, while glutamatergic PV neurons were not, confirming previously published findings (Webster et al., 2020) using different mRNA in situ hybridization methods. Further, we found that the percentage of *vgat*-positive PV neurons was significantly higher in the lateral LHb than in the medial LHb (Fig. 3*D*2; Fig. 3-1), although *vgat*-positive PV neurons were minimal in the LHb. The *vglut2*-positive PV neurons were broadly and evenly distributed in the medial and lateral LHb and were not segregated (Fig. 3*A*,*B*1–3,*C*1–3,*D*1).

In summary, in the present study, we revealed that the subsets of PV neurons were topographically organized inside the LHb anteroposteriorly, with anterior GABAergic and posterior glutamatergic PV neurons, in addition to mediolaterally, with lateral GABAergic PV neurons. These findings suggest that LHb PV neurons play distinct roles in different parts of the LHb, leading to its topographic function. However, this study had some limitations. Firstly, whether the subsets of LHb PV neurons differ between the sexes, whether the subsets are organized in the LHb subnuclei, or whether the topographic distribution of the subsets is related to the LHb subnuclei and local circuits all currently remain unclear. Additionally, it would be of interest to determine whether topography is differentially involved in various cognitive and motivational processes associated with the LHb, especially the involvement of posterior glutamatergic PV neurons.

## Synthesis

Reviewing Editor: Tod E Kippin, University of California Berkeley Decisions are customarily a result of the Reviewing Editor and the peer reviewers coming together and discussing their recommendations until a consensus is reached. When revisions are invited, a fact-based synthesis statement explaining their decision and outlining what is needed to prepare a revision will be listed below. The following reviewer(s) agreed to reveal their identity: Alessandro Castorina.

Two independent reviewers evaluated the manuscript and both emphasized many strength of the study with only a few minor concerns remaining. I believe this is very close to acceptable so please attend to the below comments in a revision.

I am available to assist if anything requires clarification as you revise your mansucript.

Minor comments

- Line 14, 15 - I would suggest to delete 'which has never been shown before'.

- Line 47 - Delete 'calcium-binding protein'.

-Line 66 and following states: "Webster et al. showed that pv mRNA-positive neurons express vgat and vglut2 mRNA (Webster et al., 2020). A[nother] previous study also showed that approximately 60% of LHb PV neurons exhibit GABA immunoreactivity (Nakamura et al., 2021). Previous [These] studies have indicated that LHb PV neurons are heterogeneous in their neurotransmitter machinery and are distributed differently in the medial and lateral LHb. However, their details have not been thoroughly elucidated."

Suggestions are in brackets [...].

- In Figures 2B and 2D 'Cortex' and 'Hipp' should be more explicitly mentioned as 'Cingulate Ctx' and 'CA3'. Densities of PV cells are known to differ between cortical regions (e.g. see PMID: 28985566; DOI: 10.1016/j.cell.2017.09.020).

- Line 398-400: "The majority of PV neurons are glutamatergic, meaning that they release glutamate from vglut2." Rephrase 'release glutamate from vglut2'. What does this mean? Release from VGLUT2-positive vesicles?

## Author Response

Responses to Reviewers The following points were corrected. All corrections and relocation of the sentences are highlighted in the manuscript.

Suggestion - Extended Data must be referenced in the legend of the table/figure it is supporting. i.e Figure 2-1 will be mentioned in the legend of Figure 2 Answer The authors referenced Extended Data in the legend of the table/figure it is supporting, as indicated.

Suggestion - Line 14, 15 - I would suggest to delete 'which has never been shown before'.

Answer The authors deleted the phrase 'which has never been shown before', as suggested.

Suggestion - Line 47 - Delete 'calcium-binding protein'.

Answer The authors deleted 'calcium-binding protein', as suggested.

Suggestion -Line 66 and following states: "Webster et al. showed that pv mRNA-positive neurons express vgat and vglut2 mRNA (Webster et al., 2020). A[nother] previous study also showed that approximately 60% of LHb PV neurons exhibit GABA immunoreactivity (Nakamura et al., 2021). Previous [These] studies have indicated that LHb PV neurons are heterogeneous in their neurotransmitter machinery and are distributed differently in the medial and lateral LHb. However, their details have not been thoroughly elucidated." Suggestions are in brackets [...].

Answer The authors corrected the words as suggested.

Suggestion - In Figures 2B and 2D 'Cortex' and 'Hipp' should be more explicitly mentioned as 'Cingulate Ctx' and 'CA3'. Densities of PV cells are known to differ between cortical regions (e.g. see PMID: 28985566; DOI: 10.1016/j.cell.2017.09.020).

Answer The authors corrected "Cortex" and "Hipp (or "hippocampus")" into "Cingulate Ctx" and "CA3 (or "hippocampus CA3")", respectively, as suggested. The abbreviations are also corrected in Figure 2B and 2D.

Suggestion - Line 398-400: "The majority of PV neurons are glutamatergic, meaning that they release glutamate from vglut2." Rephrase 'release glutamate from vglut2'. What does this mean? Release from VGLUT2-positive vesicles? Answer The authors corrected the sentence as suggested.
