# Supplementary figures and images for: Topographic Organization of Glutamatergic and GABAergic Parvalbumin-Positive Neurons in the Lateral Habenula
Source: eNeuro. 2024 Jul 12;11(7):ENEURO.0069-24.2024. doi: 10.1523/ENEURO.0069-24.2024 (PMC11255393; doi:10.1523/ENEURO.0069-24.2024)

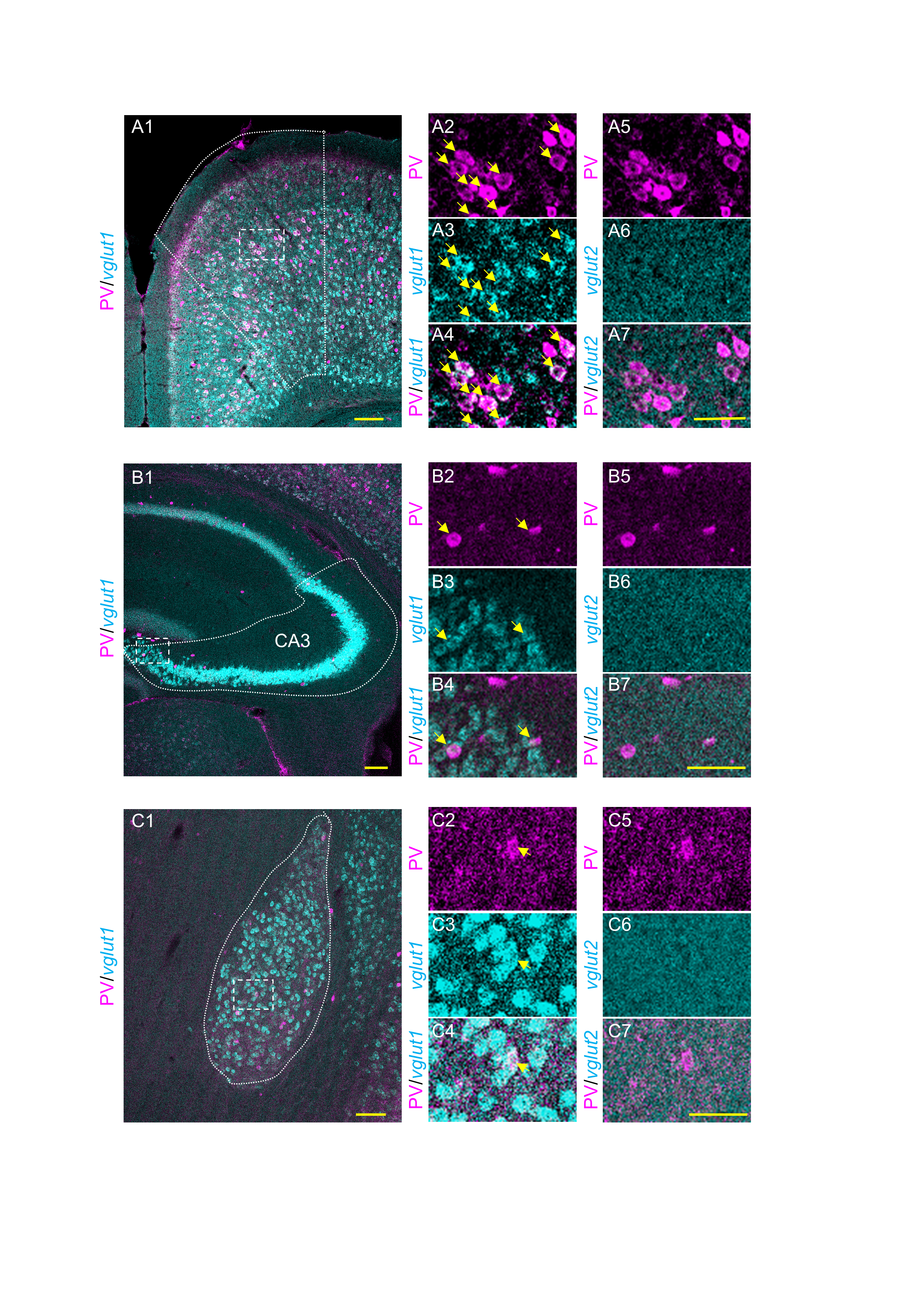

Supplement: Figure 2-1 — Heterogeneity of PV neurons in the expression of glutamatergic markers in the cingulate cortex, the hippocampus CA3, and the basolateral amygdala. A The expression of glutamatergic machinery in PV neurons in the cingulate cortex. vglut1 (cyan) and PV (magenta) are double-stained with HCR and IHC, respectively (A1). A2-7 shows the dotted square area of A1. PV neurons (magenta in A2) and expression of vglut1 (cyan in A3) are shown (merged in A4). PV neurons (magenta in A5) and expression of vglut2 (cyan in A6) are shown (merged in A7). Arrows indicate double-positive for PV and vglut1. B The expression of glutamatergic machinery in PV neurons in the hippocampus (CA3 area). vglut1 (cyan) and PV (magenta) are double-stained with HCR and IHC, respectively (B1). B2-7 shows the dotted square area of B1. PV neurons (magenta in B2) and expression of vglut1 (cyan in B3) are shown (merged in B4). PV neurons (magenta in B5) and expression of vglut2 (cyan in B6) are shown (merged in B7). Arrows indicate double-positive for PV and vglut1. C Expression of glutamatergic machinery in PV neurons in the basolateral amygdala. vglut1 (cyan) and PV (magenta) are double-stained with HCR and IHC, respectively (C1). C2-7 shows the dotted square area of C1. PV neurons (magenta in C2) and expression of vglut1 (cyan in C3) are shown (merged in C4). PV neurons (magenta in C5) and expression of vglut2 (cyan in C6) are shown (merged in C7). Arrows indicate double-positive for PV and vglut1. All images are single optical sections of confocal images. Scale bars: 100 µm (A1, B1, C1), 50 µm (A2-7, B2-7, C2-7). PV, parvalbumin. vglut1, vesicular glutamate transporter 1. vglut2, vesicular glutamate transporter 2. Download Figure 2-1, TIF file. [file eneuro-11-ENEURO.0069-24.2024-s002.tif]

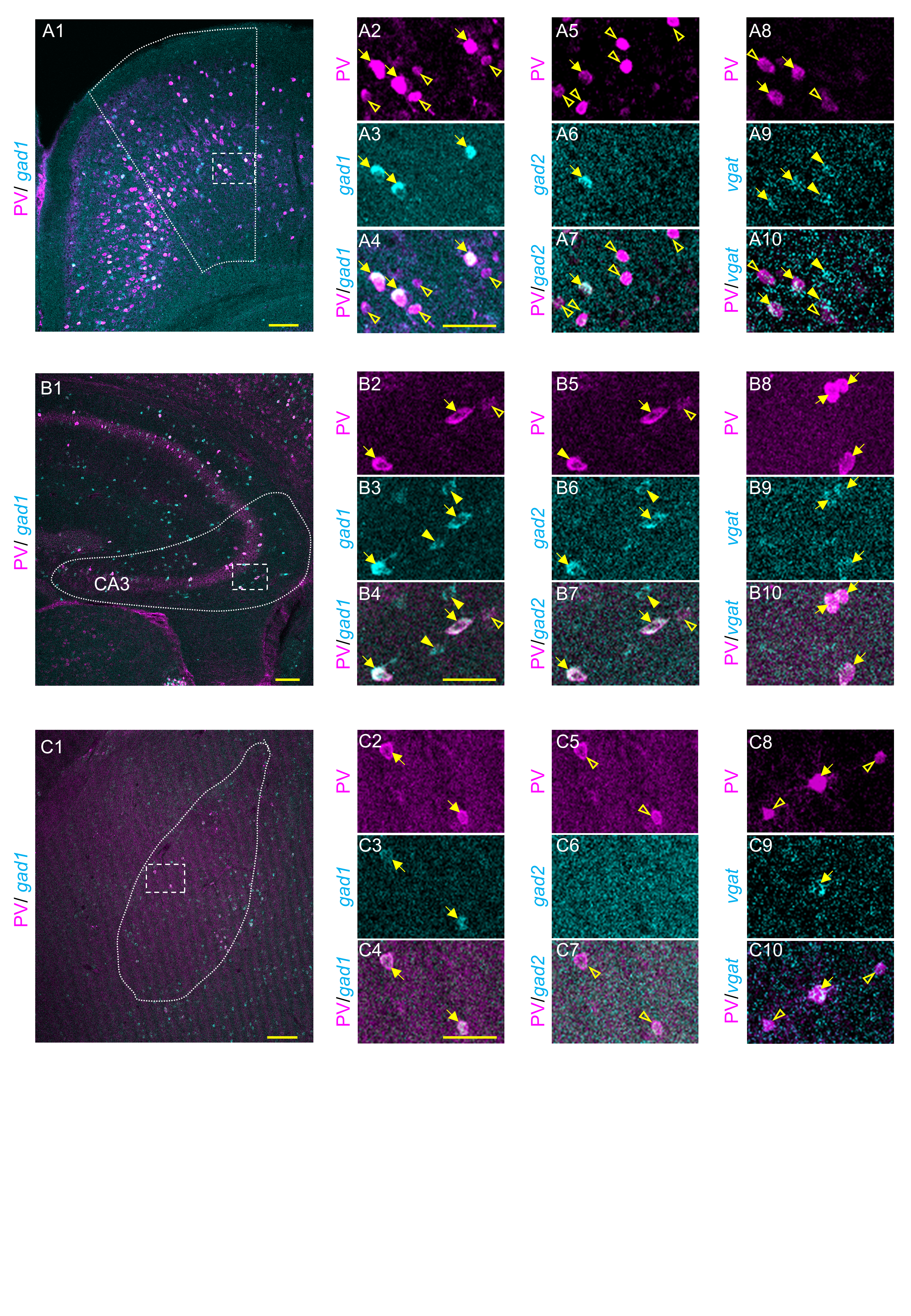

Supplement: Figure 2-2 — Heterogeneity of PV neurons in the expression of GABAergic markers in the cingulate cortex, hippocampus CA3, and basolateral amygdala. A The expression of GABAergic machinery in PV neurons in the cingulate cortex. gad1 (cyan) and PV (magenta) are double-stained with HCR and IHC, respectively (A1). A2-4 shows the dotted square area of A1. PV neurons (magenta in A2) and expression of gad1 (cyan in A3) are shown (merged in A4). PV neurons (magenta in A5) and expression of gad2 (cyan in A6) are shown (merged in A7). PV neurons (magenta in A8) and expression of vgat (cyan in A9) are shown (merged in A10). Open arrowheads indicate PV neurons. Arrows indicate positive for gad1, gad2, or vgat in PV neurons. Closed arrowheads indicate vgat positive neurons. B The expression of GABAergic machinery in PV neurons in the hippocampus (CA3 area). gad1 (cyan) and PV (magenta) are double-stained with HCR and IHC, respectively (B1). B2-4 shows the dotted square area of B1. PV neurons (magenta in B2) and expression of gad1 (cyan in B3) are shown (merged in B4). PV neurons (magenta in B5) and expression of gad2 (cyan in B6) are shown (merged in B7). PV neurons (magenta in B8) and expression of vgat (cyan in B9) are shown (merged in B10). Open arrowheads indicate PV neurons. Arrows indicate positive for gad1, gad2, or vgat in PV neurons. Closed arrowheads indicate gad1 or gad2 positive neurons. C The expression of GABAergic machinery in PV neurons in the basolateral amygdala. gad1 (cyan) and PV (magenta) are double-stained with HCR and IHC, respectively (C1). C2-4 shows the dotted square area of C1. PV neurons (magenta in C2) and expression of gad1 (cyan in C3) are shown (merged in C4). PV neurons (magenta in C5) and expression of gad2 (cyan in C6) are shown (merged in C7). PV neurons (magenta in C8) and expression of vgat (cyan in C9) are shown (merged in C10). Open arrowheads indicate PV neurons. Arrows indicate positive for gad1 or vgat in PV neurons. All images are single optical se [file eneuro-11-ENEURO.0069-24.2024-s003.tif]

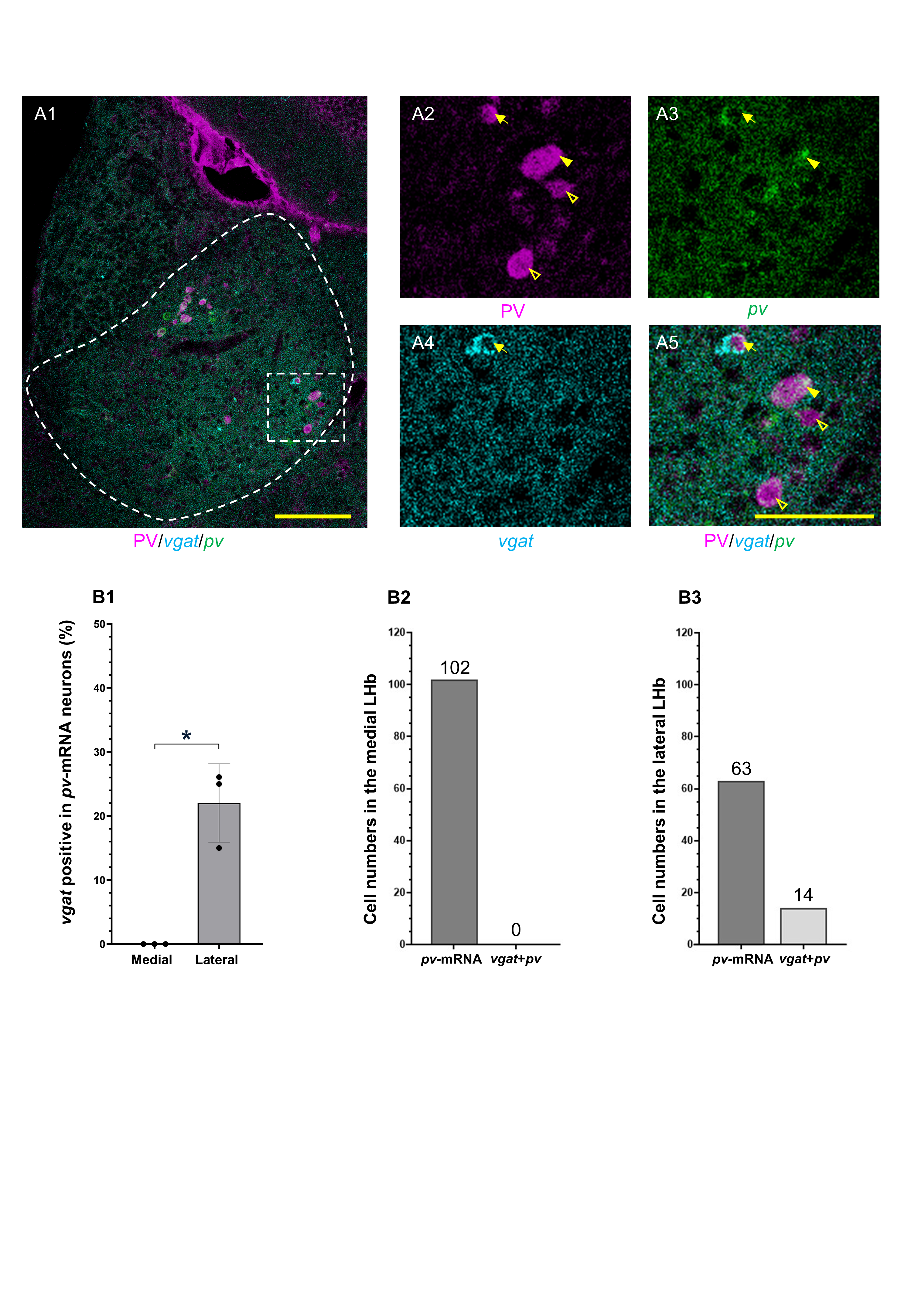

Supplement: Figure 3-1 — Mediolateral distribution of GABAergic pv mRNA positive neurons in the LHb. A PV-protein (magenta), pv-mRNA(green), and vgat (cyan) are triple-stained with IHC and HCR in the LHb (A1). The dotted square area of A1 is shown in A2-5. In PV-protein positive neurons (magenta in A2), pv-mRNA positive neurons (green in A3), and expression of vgat (cyan in A4) is shown (merged in A5). Open arrowheads indicate PV-protein positive neurons. Arrows indicate triple-positive for PV-protein, pv-mRNA, and vgat. Closed arrowheads indicate pv-mRNA positive neurons. B Percentages of vgat expression in the pv-mRNA positive neurons are compared between the medial and lateral LHb (B1). The pv-mRNA positive neurons are counted, and the vgat positive neurons among them are counted in the medial (B2) and lateral LHb (B3). The percentages of vgat positive neurons in the pv-mRNA positive neurons were significantly higher in the lateral LHb than those in the medial (vgat: 22.03 ± 3.53% in lateral and 0 ± 0% in medial, p = 0.02, Welch's t-test, HCR, B1). In the medial LHb, 102 pv-mRNA positive neurons were observed; among them, no neurons expressed vgat (B2). In contrast, 63 pv-mRNA positive neurons were observed; among them, the 14 neurons expressed vgat in the lateral LHb (B3). All images are single optical sections. Scale bars: 100 µm (A1), 50 µm (A2-5). LHb, lateral habenular nucleus. PV, parvalbumin (protein). pv, parvalbumin (mRNA). vgat, vesicular GABA transporter. N = 3 mice. *p < 0.05. Welch's t-test. Download Figure 3-1, TIF file. [file eneuro-11-ENEURO.0069-24.2024-s005.tif]
